# ﻿Morphological and molecular identification of new species and records of *Daldinia* (Hypoxylaceae, Xylariales) from Guizhou Province, China

**DOI:** 10.3897/mycokeys.123.160960

**Published:** 2025-10-16

**Authors:** Yingying Wu, Shuji Li, Ning Jiang, Chengming Tian

**Affiliations:** 1 The Key Laboratory for Silviculture and Conservation of the Ministry of Education, Beijing Forestry University, Beijing 100083, China Beijing Forestry University Beijing China; 2 Key Laboratory of Biodiversity Conservation of National Forestry and Grassland Administration, Ecology and Nature Conservation Institute, Chinese Academy of Forestry, Beijing 100091, China Ecology and Nature Conservation Institute, Chinese Academy of Forestry Beijing China

**Keywords:** Ascomycota, morphology, new taxon, phylogeny, taxonomy

## Abstract

Members of *Daldinia* are widely distributed and commonly found on decaying wood, branches, and diseased leaves. In this study, four strains of *Daldinia* were isolated from diseased leaves of *Indocalamus hirsutissimus* and *Rubus idaeus* in Guizhou Province, China. Species identification was conducted using combined nuc rDNA ITS1-5.8S-ITS2 (ITS), partial sequences of the large subunit (28S), RNA polymerase II subunit 2 (*rpb*2), and beta-*tub*ulin (*tub*2) sequence data, along with morphological comparisons. We introduce the new species *D. rubi* and *D. eschscholtzii*, accompanied by a new host record on *Indocalamus hirsutissimus*, supported by both morphological features and molecular evidence. In addition, a comprehensive analysis of *D. eschscholtzii*’s worldwide host distribution revealed that it spans 56 plant families across 31 countries. This study enhances our understanding of the species diversity of *Daldinia* and its broad host range, providing a new perspective for taxonomic research.

## ﻿Introduction

*Daldinia* was originally introduced by Cesati and De Notaris (Cesati and De Notaris 1863). In the early 20^th^ century, Child (1932) conducted the first comprehensive global study on *Daldinia*, describing 13 species and introducing anamorphic features and physiological traits for classification purposes. However, her work contained inaccuracies in species descriptions, leading to confusion. Later, Ju et al. (1997) conducted the second global revision, clarifying some errors and providing detailed descriptions of *D. bakeri*, *D. caldariorum*, and *D. loculata*. Rogers et al. (1999) redefined the type species *D. concentrica* and held that it was actually *D. childiae*. In the early 21^st^ century, the integration of molecular biology and chemotaxonomic techniques significantly advanced the taxonomic study of *Daldinia*. Johannesson et al. (2000) and Stadler et al. (2001a) constructed preliminary phylogenetic relationships using nuc rDNA ITS sequence analysis. The application of HPLC (high-performance liquid chromatography) and scanning electron microscopy (SEM) enabled the examination of chemical profiles and ultrastructures of historical specimens, revealing the diversity of secondary metabolites and their taxonomic significance in *Daldinia* (Stadler et al. 2001b, c, 2002; Bitzer et al. 2007). Subsequent ecological studies uncovered its endophytic lifestyle and rapid colonization ability following host damage (Rogers 2000; Stadler 2011). Stadler et al. (2014) integrated morphological, molecular phylogenetic, and chemotaxonomic approaches, analyzing data from thousands of specimens and cultures to elucidate the complex diversity of secondary metabolites and phylogenetic relationships in *Daldinia*. Overall, the taxonomy of *Daldinia* has evolved from a morphology-based approach to a multidisciplinary research framework, gradually resolving earlier taxonomic confusions and providing a foundation for understanding its ecological and evolutionary relationships.

In recent years, the classification framework of *Daldinia* has stabilized. Wendt et al. (2018) confirmed through multi-gene phylogenetic analysis (ITS, 28S, *rpb*2, and *tub*2) that *Daldinia* and its relatives form a distinct clade within Hypoxylaceae, clearly differentiated from *Hypoxylon* and *Pyrenopolyporus*. The application of a polyphasic approach has significantly increased species diversity, revealing multiple cryptic species. For instance, Stadler et al. (2004) described five new species from the Canary Islands, Channel Islands, and Sicily, while Wongkanoun et al. (2019) reported a new record and a new species from northern Thailand. Additionally, Pažoutová et al. (2013) identified *D. hawksworthii*, an insect-associated endophytic species, based on molecular data and conidial structures. Yin et al. (2024) isolated and identified seven new anamorphic species from diseased leaves in southern China, analyzing their geographical distribution and host specificity, which suggests that warm, humid, and vegetatively rich regions may harbor more *Daldinia* and fungal resources.

The stromata of *Daldinia* fungi are prominent and persistent, often forming dense clusters on woody plants, making them easily noticeable. Internally, the stromata exhibit horizontal zonation, a key feature distinguishing this genus from other pyrenomycete fungi (Stadler et al. 2014). Traditionally, *Daldinia* was considered a saprophyte, primarily causing white rot on dead angiosperm wood. However, recent studies suggest they may persist as endophytes within host tissues, forming stromata only when the host is damaged or stressed, leading to their previous misidentification as “rare” despite potentially widespread distribution (Stadler and Hellwig 2005). In this paper, during the identification of plant pathogenic fungi from diseased leaves in Guizhou Province, China, two *Daldinia* species were unexpectedly isolated from *Indocalamus hirsutissimus* and *Rubus idaeus*. Molecular and morphological analyses confirmed the establishment of a new species, *D. rubi* sp. nov., and reported a new host record species, *D. eschscholtzii*. This study provides detailed descriptions, illustrations, and DNA-based phylogenetic analyses to validate their taxonomic classification.

## ﻿Materials and methods

### ﻿Sample collection and fungal isolation

Diseased leaves of *Indocalamus hirsutissimus* and *Rubus idaeus* were sampled from Guizhou, China, and sampling information was recorded (Rathnayaka et al. 2025). For sampling positions on the leaf, the junction of diseased and healthy tissue was targeted. The diseased leaf tissues were cut into small pieces of approximately 5 mm × 5 mm using a sterile scalpel. These tissue pieces were then subjected to surface sterilization (rinsed in 75% ethanol for 30 s, followed by 2% sodium hypochlorite for 2 min, and finally rinsed three times in sterile distilled water). The surface-sterilized tissues were plated onto potato dextrose agar (PDA), which consisted of 20% diced potatoes, 2% agar, and 2% glucose. Petri dishes were incubated at 25 °C in the dark for 2–3 days. After colony formation, hyphal tips were carefully picked under a stereomicroscope and transferred to fresh PDA plates to obtain pure cultures, following the method described by Crous et al. (2019a). Type specimens of the new species were deposited in the Museum of Beijing Forestry University (BJFC), and ex-type living cultures were preserved at the China Forestry Culture Collection Center (CFCC), Beijing, China.

### ﻿Morphological observation

Cultures were grown on PDA at 25 °C under a 12 h light/dark cycle (Crous et al. 2019b). After 14 days, colony measurements were taken, and characteristics such as color, shape, and aerial mycelium density were observed and recorded. Slide mounts were prepared in water from sporulating colonies on PDA. Observations were performed using a LEICA DM 2500 dissecting microscope (Wetzlar, Germany) and a NIKON ECLIPSE 80i compound microscope with differential interference contrast (DIC) illumination. Images were captured using a NIS DS-RI2 camera with Nikon NIS-Elements F4.30.01 software. Conidial length was measured from the base of the basal cell to the base of the apical appendage, and conidial width was measured at the widest point. A random selection of 50 conidia was used for measurements. Taxonomic novelties were deposited in MycoBank (Crous et al. 2004).

### ﻿DNA extraction, PCR amplification, and sequencing

When mycelia spread fully on PDA, genomic DNA was extracted using the cetyltrimethylammonium bromide (CTAB) method. PCR primers (forward and reverse) and amplification conditions are detailed in Table 1. PCR amplification was performed on a BIO-RAD PTC-200 thermal cycler. Each 20 μL reaction system contained 10 μL of Master Mix (Promega Corporation), 7 μL of double-deionized water, 1 μL each of forward and reverse primers, and 1 μL of DNA template. PCR products were analyzed by 2% agarose gel electrophoresis and sent to Tsingke Biotechnology Co., Ltd. (Beijing, China) for sequencing.

**Table 1.** Genes used in this study with PCR primers.

Locus	PCR primers	PCR: thermal cycles (annealing temp. in bold)	References
ITS	ITS1/ITS4	(95 °C: 30 s, 51 °C: 30 s, 72 °C: 1 min) × 35 cycles	White et al. 1990
28S	LROR/LR5	(95 °C: 45 s, 55 °C: 30 s, 72 °C: 1 min) × 35 cycles	Vilgalys and Hester 1990
*rpb2*	fRPB2-5F/fRPB2-7cR	(95 °C: 15 s, 55 °C: 30 s, 72 °C: 1 min) × 35 cycles	Liu et al. 1999
*tub2*	T1/T22	(95 °C: 35 s, 52 °C: 55 s, 72 °C: 2 min) × 35 cycles	O’Donnell and Cigelnik 1997

### ﻿Phylogenetic analyses

The sequences obtained were assembled using SEQMAN software, and reference sequences from related publications (Yin et al. 2024; Liu et al. 2025) were retrieved from the National Center for Biotechnology Information (NCBI; https://www.ncbi.nlm.nih.gov). All sequences generated in this study were submitted to GenBank (Table 2). Sequences were aligned in MAFFT on the web server (https://mafft.cbrc.jp/alignment/server/) (Katoh and Standley 2013; Katoh et al. 2019), and further adjustments and editing were made with MEGA (Tamura et al. 2013). The multigene sequence alignments and the resulting trees were deposited in TreeBASE (https://treebase.org; study ID S32158). Maximum parsimony (MP), maximum likelihood (ML), and Bayesian inference (BI) were selected to construct phylogenetic trees using PAUP, PHYML, and MRBAYES (Huelsenbeck and Ronquist 2001; Swofford 2003; Silvestro and Michalak 2012). Phylograms were visualized with FIGTREE (http://tree.bio.ed.ac.uk/software/figtree/) and further edited with ADOBE ILLUSTRATOR CS (Adobe Systems Inc., USA). Maximum-parsimony bootstrap values (MPBP) and maximum-likelihood bootstrap values (MLBP) ≥ 50% and Bayesian posterior probabilities (BYPP) ≥ 0.90 were shown on the tree.

**Table 2.** Information on strains used in phylogenetic analysis of the genus *Daldinia*.

Species	Strains	Country	GenBank Accession Numbers			
			ITS	28S	rpb2	tub2
*Annulohypoxylon annulatum*	CBS 140775	USA	KY610418	N/A	KY624263	KX376353
*A. moriforme*	CBS 123579	France	KX376321	KY610425	KY624289	KX271261
*A. nitens*	MFLUCC 12.0823	Thailand	KJ934991	KJ934992	KJ934994	KJ934993
*A. truncatum*	CBS 140778	USA	KY610419	N/A	KY624277	KX376352
*Daldinia analina*	CBS 114736^T^	Ecuador	AM749918	KY610430	KY624239	KC977259
*D. bambusicola*	CBS 122872^T^	Thailand	KY610385	KY610431	KY624241	AY951688
*D. bambusicola*	TBRC 8878	Thailand	MH922869	MH922870	MK165431	MK165422
*D. bambusicola*	TBRC 8879	Thailand	MH922872	MH938543	MK165432	MK165423
*D. bambusicola*	BCC33678	Thailand	MN153860	MN153877	MN172218	N/A
*D. brachysperma*	BCC33676	Thailand	MN153854	MN153871	N/A	MN172205
*D. caldariorum*	MUCL 49211	France	AM749934	KY610438	KY624242	KC977282
*D. caldariorum*	CBS 122874	USA	KU683756	KU683796	KU684289	KU684128
*D. chiangdaoensis*	BCC88202^T^	Thailand	MN153850	MN153867	MN172208	MN172197
*D. chiangdaoensis*	BCC88221	Thailand	MN153851	MN153868	MN172209	MN172198
*D. concentrica*	CBS 113277	Germany	AY616683	KY610434	KY624243	KC977274
*D. dennisi*	CBS 114741^T^	Australia	JX658477	KY610435	KY624244	KC977262
*D. ehretiae*	SAUCC228302^T^	China	PP145319	PP198888	PP263613	PP277051
*D. ehretiae*	SAUCC228303	China	PP145320	PP198889	PP263614	PP277052
D. eschscholtzii	CFCC 72597	China	PV565503	PV548037	N/A	PV649844
D. eschscholtzii	CFCC 72598	China	PV565504	PV548038	N/A	PV649845
*D. eschscholtzii*	MUCL 45435	Benin	JX658484	KY610437	KY624246	KC977266
*D. eschscholtzii*	TRRC 8876	Thailand	MH938532	MH938541	MK165429	MK165420
*D. eschscholtzii*	BCC28017	Thailand	MN153862	MN153879	MN172215	N/A
*D. eschscholtzii*	BCC62428	Thailand	MN153863	MN153880	MN172216	N/A
*D. flavogranulata*	BCC89363^T^	Thailand	MN153856	MN153873	MN172211	MN172200
*D. flavogranulata*	BCC89365	Thailand	MN153857	MN153874	MN172212	MN172201
*D. flavogranulata*	BCC89376	Thailand	MN153858	MN153875	MN172213	MN172202
*D. guizhouensis*	GMB0719^T^	China	PQ884703	PQ885415	PQ893623	PQ893600
*D. guizhouensis*	GMB5611	China	PQ884704	PQ885416	PQ893624	PQ893601
*D. jianfengensis*	SAUCC373804^T^	China	PP145325	PP198890	PP263615	PP277053
*D. jianfengensis*	SAUCC373805	China	PP145326	PP198891	PP263616	PP277054
*D. korfii*	EBS067	Argentina	KY204018	N/A	N/A	KY204014
*D. korfii*	EBS473	Argentina	KY204020	N/A	N/A	KY204016
*D. kretschmaroides*	TBRC 8875	Thailand	MH938531	MH938540	MK165425	MK165416
*D. ledongensis*	SAUCC393602^T^	China	PP145327	PP198892	N/A	PP277055
*D. ledongensis*	SAUCC393603	China	PP145328	PP198893	N/A	PP277056
*D. loculatoides*	CBS 113279	UK	AF176982	KY610438	KY624247	N/A
*D. macaronesica*	CBS 113040	Spain	KY610398	KY610477	KY624294	N/A
*D. menghaiensis*	SAUCC242404^T^	China	PP145323	PP198894	PP263617	PP277057
*D. menghaiensis*	SAUCC242405	China	PP145324	PP198895	PP263618	PP277058
*D. petriniae*	MUCL 49014	Austria	AM749937	KY610439	KY624248	N/A
*D. phadaengensis*	BCC89349^T^	Thailand	MN153852	MN153869	MN172206	MN172195
*D. phadaengensis*	BCC89359	Thailand	MN153853	MN153870	MN172207	MN172196
*D. pyrenaica*	MUCL 53969	France	KY610413	N/A	KY624274	N/A
*D. rhododendri*	SAUCC460001^T^	China	PP145330	PP198896	N/A	PP277059
*D. rhododendri*	SAUCC460002	China	PP145329	PP198897	N/A	PP277060
D. rubi	CFCC 72599^T^	China	PV565505	PV548039	N/A	PV649846
D. rubi	CFCC 72600	China	PV565506	PV548040	N/A	PV649847
*D. spatholobi*	SAUCC203501^T^	China	PP145318	PP198898	N/A	PP277061
*D. spatholobi*	SAUCC203502	China	PP145317	PP198899	N/A	PP277062
*D. steglicilii*	MUCL 43512	Papua New	KY610399	KY610479	KY624250	N/A
*D. subvernicosa*	TBRC 8877^T^	Thailand	MH938533	MH938542	MK165430	MK165421
*D. theissenii*	CBS 113044	Argentina	KY610388	KY610441	KY624251	N/A
*D. thunbergiae*	SAUCC228601^T^	China	PP145322	PP198900	N/A	PP277063
*D. thunbergiae*	SAUCC228602	China	PP145321	PP198901	N/A	PP277064
*D. vernicosa*	CBS 119316	Germany	KY610395	KY610442	KY624252	N/A
*Hypomontagnella monticulosa*	MUCL 54604	French Guiana	KY610404	KY610487	KY624305	KX271273
*H. submonticulosa*	CBS 115280	France	KC968923	KY610457	KY624226	KC977267
*Hypoxylon fragiforme*	MUCL 51264	Germany	KC477229	KM186295	KM186296	KX271282
*Hy. fuscum*	CBS 113049	France	KY610401	KY610482	KY624299	KX271271
*Hy. haematostroma*	MUCL 53301	Martinique	KC968911	KY610484	KY624301	KC977291
*Jackrogersella cohaerens*	CBS 119126	Germany	KY610396	KY610497	KY624270	KY624314
*J. minutella*	CBS 119015	Portugal	KY610381	KY610424	KY624235	KX271240
*Pyrenopolyporus hunteri*	MUCL 52673^T^	Ivory Coast	KY610421	KY610472	KY624309	KU159530
*P. laminosus*	TBRC 8871	Thailand	MH938527	MH938536	MK165424	MK165415
*P. laminosus*	MUCL 53305^T^	Martinique	KC968934	KY610485	KY624303	KC977292
*P. nicaraguensis*	CBS 117739^T^	Burkina Faso	AM749922	KY610489	KY624307	KC977272
*Rostrohypoxylon terebratum*	CBS 119137^T^	Thailand	DQ631943	DQ840069	DQ631954	DQ840097
*Xylaria arbuscula*	CBS 126415	Germany	KY610394	KY610463	KY624287	KX271257
*X. brunneovinosa*	HAST 720	Martinique	EU179862	N/A	GQ853023	GQ502706

Notes: Strains in this study are marked in bold. “T”: ex-type strains. N/A = not available.

Maximum parsimony (MP) analysis used the tree bisection and reconnection (TBR) branch-swapping algorithm with 1,000 random-addition sequences in a heuristic search (Swofford 2003). The maximum number of trees was set at 5,000 branches of zero length, and all parsimonious trees were saved. Tree length (TL), consistency index (CI), retention index (RI), and rescaled consistency index (RC) were calculated (Swofford 2003). For maximum likelihood (ML) analysis, the GTR GAMMA model of site substitution was applied, estimating gamma-distributed rate heterogeneity and the proportion of invariant sites (Guindon et al. 2010). Branch support in MP and ML was evaluated using a 1,000-replicate bootstrap (BS) method (Hillis and Bull 1993). Bayesian inference (BI) analysis, using a Markov chain Monte Carlo (MCMC) algorithm, was employed to calculate Bayesian posterior probabilities (Rannala and Yang 1996). MRMODELTEST (Posada and Crandall 1998) was used to estimate the nucleotide substitution model for weighted Bayesian analysis. Two MCMC chains, starting from random trees, were run for 1,000,000 generations until the average standard deviation of split frequencies dropped below 0.01, sampling trees every 100^th^ generation. The first 25% of trees were discarded as burn-in, and Bayesian posterior probabilities (BPP) were calculated from the remaining 7,500 trees.

## ﻿Result

### ﻿Phylogenetic analyses

**Figure 1.**
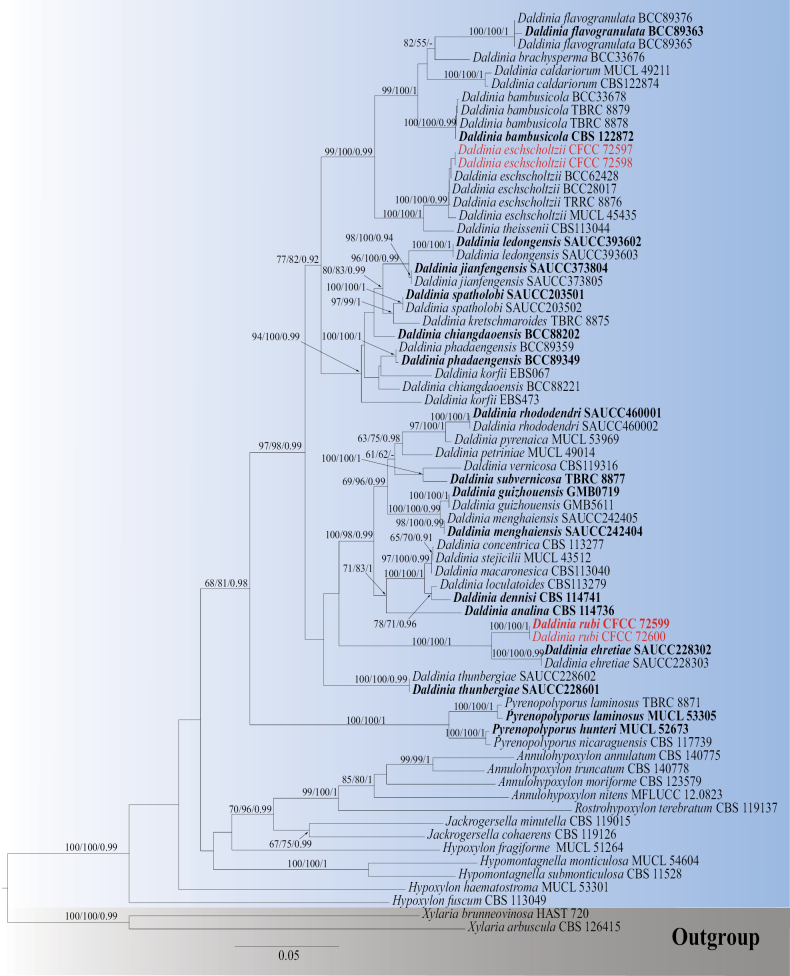
Phylogram generated from RAxML analysis based on ITS, 28S, *rpb*2, and *tub*2 sequence data of *Daldinia* isolates. The tree was rooted with *Xylaria brunneovinosa* (HAST 720) and *X. arbuscula* (CBS 126415). MP, ML (≥ 50%), and BI (≥ 0.9) bootstrap supports are shown near the nodes. Isolates from this study are marked in red, and ex-type strains are marked in bold.

The BLAST results indicated that the four isolates belong to *Daldinia*. In this genus, the combined ITS, 28S, *rpb*2, and *tub*2 dataset consisted of 3,145 characters, including alignment gaps (408 for ITS, 801 for 28S, 830 for *rpb*2, and 1,106 for *tub*2), of which 2,006 were constant and 228 were variable but parsimony-uninformative characters. MP analysis with the remaining 911 parsimony-informative characters resulted in one equally parsimonious tree: tree length (TL) = 4,936; consistency index (CI) = 0.369; retention index (RI) = 0.682; and rescaled consistency index (RC) = 0.251. In ML analysis based on the combined gene dataset, the matrix had 239 distinct alignment patterns. Estimated base frequencies were as follows: A = 0.238203, C = 0.267126, G = 0.257477, T = 0.237194, AC = 0.732392, AG = 5.679375, AT = 0.806583, CG = 0.787827, CT = 4.670947, GT = 1.000000, gamma distribution shape parameter α = 0.178803, and likelihood value ln = −26014.402666. The phylogenetic analysis revealed that the four isolates (CFCC 72597, CFCC 72598, CFCC 72599, CFCC 72600) were categorized into two clades, representing one new species, *D. rubi*, and one known species, *D. eschscholtzii* (Fig. 1). The single-gene trees for ITS, 28S, and *tub*2 of *Daldinia* are shown in Suppl. material 1.

### ﻿Taxonomy

#### ﻿ Daldinia eschscholtzii

Taxon classificationFungiXylarialesHypoxylaceae

(Ehrenb.: Fr.) Rehm, Annls mycol. 2(2): 175. 1904.

06AB00A0-0394-5B4C-A691-DA9707D1738B

858704

Fig. 2

##### Description.

Sexual morph: not observed. Asexual morph: **Conidiophores** with *Virgariella*-like to *Nodulisporium*-like branching, mononematous or dichotomous, bearing 1–4 conidiogenous cells per terminus, smooth to finely roughened, hyaline, aseptate, 23.5–28.5 × 2.5–3 (av. ± S.D.= 26 ± 1.9 × 3 ± 0.2 µm, n = 30) μm. **Conidiogenous cells** terminal or lateral, cylindrical to phialidic, smooth-finely roughened, hyaline, aseptate, apical, 9–14.5 × 2–3 μm (av. ± S.D.= 11.5 ± 1.5 × 2.5 ± 0.3 µm, n = 30). **Conidia** ellipsoid, cylindrical, oval in shape, smooth, hyaline to pale yellow, aseptate, solitary, holoblastic-sympodial, 4.5–6.5 × 2–4 μm (av. ± S.D.= 5.5 ± 0.5 × 3.0 ± 0.4 µm, n = 50).

##### Culture characters.

Colonies were dense and uniform, with entire margins and velvety texture, fluffy, surface grayish-white, slightly darker centrally, reverse pale yellow to buff, dark brown at the center. Colonies developed abundant aerial hyphae and reached 60 mm in diameter after 7 days on PDA at 25 °C.

**Figure 2.**
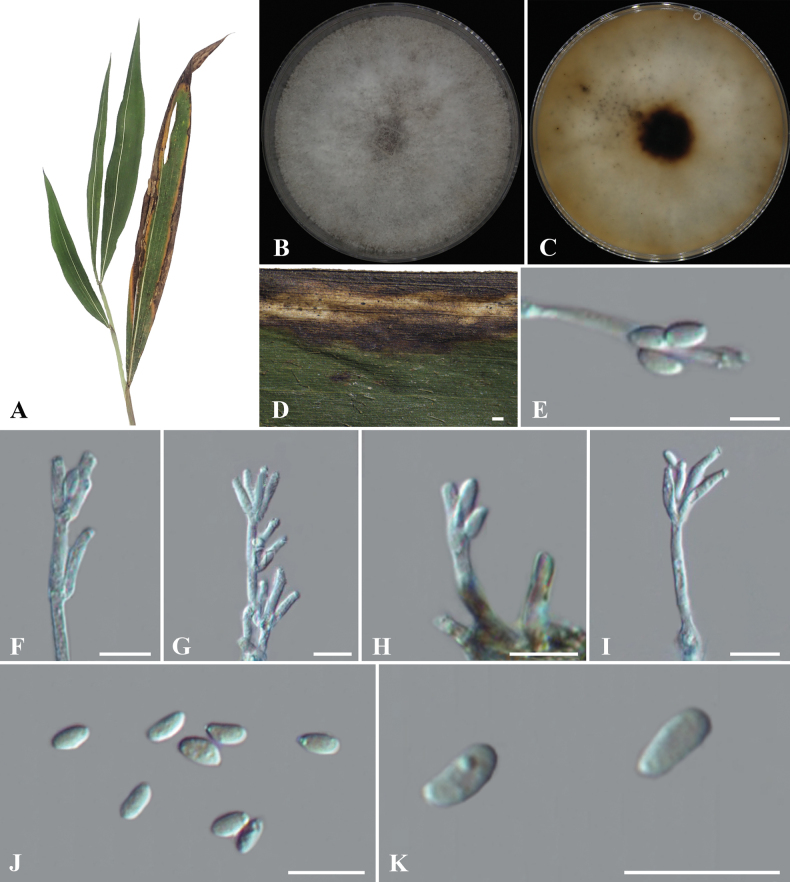
*Daldinia eschscholtzii* (BJFC-S2541) A, D. Leaf of host *Rubus idaeus*; B. Front colony morphology on PDA at 14 days; C. Reverse colony morphology on PDA at 14 days; E–I. Conidiogenous cells and conidia; J, K. Conidia. Scale Bars: 500 μm (D); 10 μm (E–K).

##### Materials examined.

China • Guizhou Province, Xingyi City, Maling River Canyon Scenic Area, 25°7'49"N, 104°57'19"E, on the leaf spots of *Indocalamus hirsutissimus*, 26 Jul 2024, C.M. Tian, N. Jiang, S.J. Li and Y.Y. Wu, BJFC-S2541, living culture CFCC 72597; *ibid.*BJFC-S2542, living culture CFCC 72598.

##### Notes.

Based on multi-locus phylogenetic analysis, two strains (CFCC 72597 and CFCC 72598) formed a highly supported clade with *Daldinia eschscholtzii* (100% MP/100% ML/0.99 BYPP). *D. eschscholtzii* is a multifunctional wood-inhabiting fungus exhibiting endophytic, saprophytic, and pathogenic traits (Stadler et al. 2014). *Daldinia eschscholtzii* exhibits endophytic, saprophytic, and pathogenic traits (Stadler et al. 2014) and has a broad host range spanning 56 plant families across 31 countries (Suppl. material 2), mainly colonizing decaying dicotyledonous wood and occasionally occurring on marine algae (Karnchanatat et al. 2007; Zhang et al. 2008; Tarman et al. 2012). Its human pathogenic potential is also confirmed (Ng et al. 2012, 2016; Yew et al. 2014; Chan et al. 2015). This study represents the first documented record of *D. eschscholtzii* on *Indocalamus*, supported by phylogenetic congruence with known *D. eschscholtzii* and alignment with its generalist ecology of colonizing lignocellulosic substrates, like *Indocalamus*.

#### ﻿ Daldinia rubi

Taxon classificationFungiXylarialesHypoxylaceae

Y.Y. Wu & C.M. Tian sp. nov.

744209AA-4038-5ED0-B724-3F97FA17E91A

858703

Fig. 3

##### Type.

China • Guizhou Province, Guiyang City, Yunyan District, Qianlingshan Forest Park, 26°36'06"N, 106°41'42"E, on the leaf spots of *Rubus idaeus*, 26 Jul 2024, C.M. Tian, N. Jiang, S.J. Li, and Y.Y. Wu (***holotype***BJFC-S2543). Ex-type culture CFCC 72599.

##### Etymology.

Named after the host genus, *Rubus*.

##### Description.

Sexual morph: not observed. Asexual morph: ***Conidiophores*** are mononematous or dichotomously branched, displaying a *Virgariella*-like to *Nodulisporium*-like branching pattern. Conidiophores smooth to finely roughened, hyaline, aseptate, with 2–3 conidiogenous cells at each terminus, measuring (14–)16–36(–39) × 2.5–4(–7) μm (av. ± S.D.= 27 ± 8.4 × 3.5 ± 1.2 µm, n = 30). ***Conidiogenous cells*** terminal or lateral, cylindrical, hyaline to pale yellow, smooth to finely roughened, with flattened base, producing conidia apically, measuring (11.5–)13–20.5 × 2–3.5 μm (av. ± S.D.= 16 ± 2.5 × 3 ± 0.4 µm, n = 30). ***Conidia*** ellipsoid to dacryoid, hyaline, aseptate, smooth to finely roughened, solitary, mostly flat-based, holoblastic-sympodial, measuring 4.5–8 × 3–4.5 μm (av. ± S.D.= 7 ± 0.8 × 4 ± 0.3 µm, n = 50).

**Figure 3.**
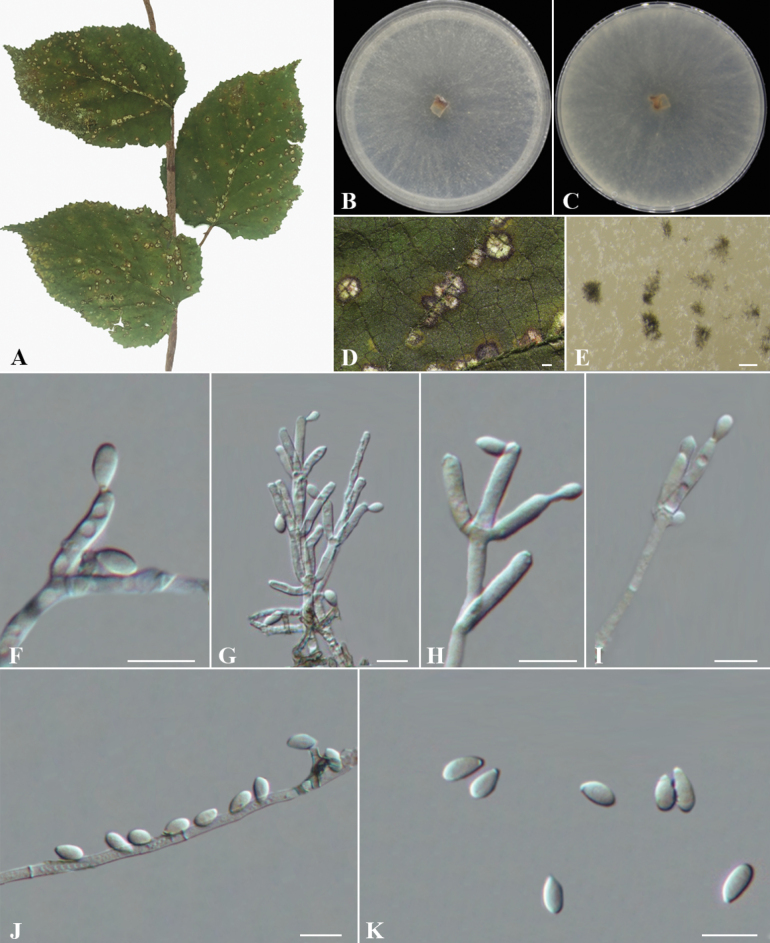
*Daldinia rubi* (BJFC-S2543) A, D. Leaf of host *Rubus idaeus*; B. Front colony morphology on PDA at 14 days; C. Reverse colony morphology on PDA at 14 days; E. Conidiomata development on medium; F–J. Conidiogenous cells and conidia; K. Conidia. Scale Bars: 500 μm (D); 200 μm (E); 10 μm (F–K).

##### Culture characters.

Colonies showed sparse, cobweb-like mycelium, appearing semi-transparent, pale gray, with small brown central structures. The reverse was pale grayish-brown with scattered black conidial masses. Aerial hyphae were sparse, growing to 60 mm on PDA in 7 days at 25 °C.

##### Other material examined.

China • Guizhou Province, Guiyang City, Yunyan District, Qianlingshan Forest Park, 26°36'06"N, 106°41'42"E, on the leaf spots of *Rubus idaeus*, 26 Jul 2024, C.M. Tian, N. Jiang, S.J. Li and Y.Y. Wu, BJFC-S2544, living cultures CFCC 72600.

##### Notes.

Based on multi-locus phylogenetic analysis, the two isolates (CFCC 72599, CFCC 72600) formed an independent clade with 100% MP, 100% ML, and 1.00 BYPP values, clearly distinct from *Daldinia ehretiae* in the multi-locus analyses (Fig. 1). To further substantiate the recognition of *D. rubi* as a new species, a comprehensive comparison of asexual morphological traits within the *Daldinia* genus in China was conducted (Table 3). Morphologically, *D. rubi* can be readily distinguished from *D. ehretiae* by multi-trait divergence: as shown in Table 3, *D. rubi* produces larger conidia (ellipsoid to dacryoid, 4.5–8 × 3–4.5 μm) compared to *D. ehretiae* (ellipsoid or cylindrical, 4.2–6.6 × 1.7–2.8 μm). For conidiophores, *D. rubi* has shorter and narrower structures (mononematous or dichotomously branched, 16–36 × 2.5–4 μm) with more conidiogenous cells per terminus (2–3 cells) than *D. ehretiae* (mononematous or dichotomously branched, 100–210 × 3.1–4.3 μm, 1–2 cells per terminus). Conidiogenous cells of *D. rubi* are shorter in length (cylindrical or laterally cylindrical, (11.5–)13–20.5 × 2–3.5 μm) versus *D. ehretiae* (cylindrical, 16.8–24.5 × 2.7–4.1 μm). Molecularly, *D. rubi* also shows clear divergence from *D. ehretiae*. There is a 12 bp difference in ITS sequences (376 characters, 96.8% similarity, including one gap) and a 26 bp difference in *tub*2 sequences (745 characters, 96.5% similarity, no gaps). Collectively, the independent phylogenetic position, distinct morphological traits (as detailed in Table 3 for a comparison of asexual characteristics among *Daldinia* species in China), and molecular divergence confirm that *D. rubi* represents a new species.

**Table 3.** Comparative analysis of asexual morphological traits among *Daldinia* species.

Species	Conidiophores (Branching/Conidiogenous Cells/Size)	Conidiogenous Cells (Shape/Size)	Conidia (Shape/Size)	References
*D. bambusicola*	Dichotomously or trichotomously branched, 2–3 conidiogenous cells per terminus, 110–160 × 2.1–2.7 μm	Cylindrical, 10.1–15.3 × 2.5–3.1 μm	Subglobose or ellipsoid, 3.4–4.5 × 2.5–3.1 μm	Yin et al. (2024)
*D. childiae*	Dichotomously or trichotomously branched, 2–3 conidiogenous cells per terminus, 150–220 × 2.5–3 μm	Clavate (apically enlarged), 14.9–32.7 × 2.8–4.4 μm	Subglobose or ellipsoid, 5.8–9.1 × 4.1–5.8 μm	Yin et al. (2024)
*D. ehretiae*	Mononematous or dichotomously branched, 1–2 conidiogenous cells per terminus, 100–210 × 3.1–4.3 μm	Cylindrical, 16.8–24.5 × 2.7–4.1 μm	Ellipsoid or cylindrical, 4.2–6.6 × 1.7–2.8 μm	Yin et al. (2024)
*D. eschscholtzii*	Mononematous, dichotomously or trichotomously branched, 2–3 conidiogenous cells per terminus, 120–214 × 2.3–4.1 μm	Cylindrical, 14.9–22.7 × 2.1–3.6 μm	Ellipsoid or dacryoid, 4.9–6.8 × 2.3–3.5 μm	Yin et al. (2024)
*D. jianfengensis*	Mononematous, dichotomously or trichotomously branched, 1–4 conidiogenous cells per terminus, 70–120 × 2.9–4.4 μm	Cylindrical, 12.1–16.9 × 2.6–3.6 μm	Subglobose or ellipsoid, 3.2–5.5 × 2.6–3.7 μm	Yin et al. (2024)
*D. ledongensis*	Rarely mononematous or dichotomously branched, 1 conidiogenous cell per terminus, 120–200 × 1.7–2.1 μm	Clavate, 8.6–15.1 × 1.2–3.4 μm	Ellipsoid or fusiform, 3.2–4.0 × 1.4–2.0 μm	Yin et al. (2024)
*D. menghaiensis*	Dichotomously or trichotomously branched (occasionally), 1–2 conidiogenous cells per terminus, 80–150 × 1.9–3.4 μm	Cylindrical or clavate, 16.9–23.5 × 2.0–3.5 μm	Ellipsoid, subglobose or dacryoid, 4.7–8.2 × 3.1–4.0 μm	Yin et al. (2024)
*D. rhododendri*	Rarely mononematous or dichotomously branched, conidiogenous cell number unclear, 40–90 × 1.4–2.0 μm	Cylindrical or ampulliform, 5.9–11.6 × 1.1–2.9 μm	Ellipsoid, cylindrical or banana - shaped, 3.2–5.1 × 1.1–2.3 μm	Yin et al. (2024)
D. rubi	Mononematous or dichotomously branched, 2–3 conidiogenous cells per terminus, 16–36 × 2.5–4 μm	Cylindrical or laterally cylindrical, (11.5–)13–20.5 × 2–3.5 μm	Ellipsoid to dacryoid, 4.5–8 × 3–4.5 μm	This study
*D. thunbergiae*	Mononematous, dichotomously or trichotomously branched, 1–4 conidiogenous cells per terminus, 70–220 × 1.8–4.1 μm	Cylindrical or clavate, 6.7–17.3 × 1.9–2.5 μm	Ellipsoid or teardrop - shaped, 3.2–5.0 × 2.2–3.1 μm	Yin et al. (2024)

## ﻿Discussion

In this study, two *Daldinia* species, *D. eschscholtzii* and *D. rubi*, were discovered on leaf spot samples of *Indocalamus hispidus* and *Rubus idaeus* in Guizhou Province, China. This represents the first record of *Daldinia* on host plants belonging to *Rubus* (Rosaceae) and *Indocalamus* (Poaceae). Globally, *Daldinia* exhibits an exceptionally broad host range, with the USDA database documenting over 600 host species (https://fungi.ars.usda.gov/). Within China, previously reported *Daldinia* species have primarily been found on woody plants of Fagaceae, Lauraceae, Sapindaceae, Moraceae, and Betulaceae (https://fungi.ars.usda.gov/). By contrast, records on Rosaceae and Poaceae hosts remain scarce. The present study significantly expands the known host range of *Daldinia* in China.

Notably, *Daldinia* exhibits distinctive biological characteristics in its sexual morph, most remarkably through the formation of large stromata on woody branches. These structures characteristically display concentric zonation patterns internally and produce ellipsoidal, brownish to dark brown ascospores (Stadler et al. 2001a; Stadler et al. 2004; Stadler et al. 2014; Liu et al. 2025). However, our investigations, consistent with previous studies, have failed to observe a sexual stage in *Daldinia* specimens collected from diseased foliage in Southwest China (Yin et al. 2024). This phenomenon suggests that the life cycle transition in *Daldinia* may be influenced by an intricate interplay of environmental conditions, climatic factors, and host-specific characteristics. Moreover, *Daldinia* may initially colonize leaves as endophytes or latent pathogens. When host vigor declines or environmental conditions become favorable, these fungi may exhibit pathogenicity. Subsequent studies should conduct artificial inoculation experiments to confirm *Daldinia*’s pathogenicity and determine how environmental factors and host conditions affect its virulence.

This study systematically compiled the global host range of *D. eschscholtzii*, revealing its ability to parasitize over 50 plant families while demonstrating distinct host preferences (Suppl. material 2). These fungi primarily colonize dicotyledonous plants, with a particular affinity for the Rosales and Fabales orders, showing the highest frequency on the Fabaceae, Lauraceae, and Moraceae families. Notably, *D. eschscholtzii* exhibits cross-clade infectivity, capable of rarely colonizing gymnosperms (Pinaceae) and ferns. As shown in Suppl. material 2, tropical-affiliated families (e.g., Lauraceae and Myrtaceae) are disproportionately represented among hosts, many of which include economically significant crops such as citrus and soybean. These characteristics reflect an evolutionary balance between broad-spectrum infectivity and specialized host adaptation in *D. eschscholtzii*.

Current studies reveal a wide distribution of *Daldinia* fungi around the world, demonstrating their strong adaptability to diverse ecological environments (Chen 2002; Stadler et al. 2014; Yin et al. 2024; Liu et al. 2025). This suggests potential undiscovered *Daldinia* species across various ecosystems, warranting more systematic investigations. Targeted sampling in special habitats (e.g., karst formations) and non-conventional hosts would particularly advance our understanding of the biodiversity and ecological functions of this genus.

## Supplementary Material

XML Treatment for Daldinia eschscholtzii

XML Treatment for Daldinia rubi

## ﻿Additional information

### Use of AI

No use of AI was reported.

### Author ORCIDs

Yingying Wu https://orcid.org/0009-0007-5095-2738

Shuji Li https://orcid.org/0009-0006-4734-8399

Ning Jiang https://orcid.org/0000-0002-9656-8500

Chengming Tian https://orcid.org/0000-0002-3352-7664

### Data availability

All of the data that support the findings of this study are available in the main text or Supplementary Information.

## Supplementary materials

Supplementary material 1 Supplementary image Data type pdf https://binary.pensoft.net/file/1441338This dataset is made available under the Open Database License (http://opendatacommons.org/licenses/odbl/1.0/). The Open Database License (ODbL) is a license agreement intended to allow users to freely share, modify, and use this Dataset while maintaining this same freedom for others, provided that the original source and author(s) are credited.Yingying Wu, Shuji Li, Ning Jiang, Chengming Tian

Supplementary material 2 Supplementary table Data type docx https://binary.pensoft.net/file/1441339This dataset is made available under the Open Database License (http://opendatacommons.org/licenses/odbl/1.0/). The Open Database License (ODbL) is a license agreement intended to allow users to freely share, modify, and use this Dataset while maintaining this same freedom for others, provided that the original source and author(s) are credited.Yingying Wu, Shuji Li, Ning Jiang, Chengming Tian
