# Supplementary material for: ﻿Morphological and molecular identification of new species and records of Daldinia (Hypoxylaceae, Xylariales) from Guizhou Province, China
Source: MycoKeys. 2025 Oct 16;123:253–69. doi: 10.3897/mycokeys.123.160960 (PMC12550505; doi:10.3897/mycokeys.123.160960)
Supplement: Supplementary material 1 — Supplementary image [file mycokeys-123-253-s001.pdf]

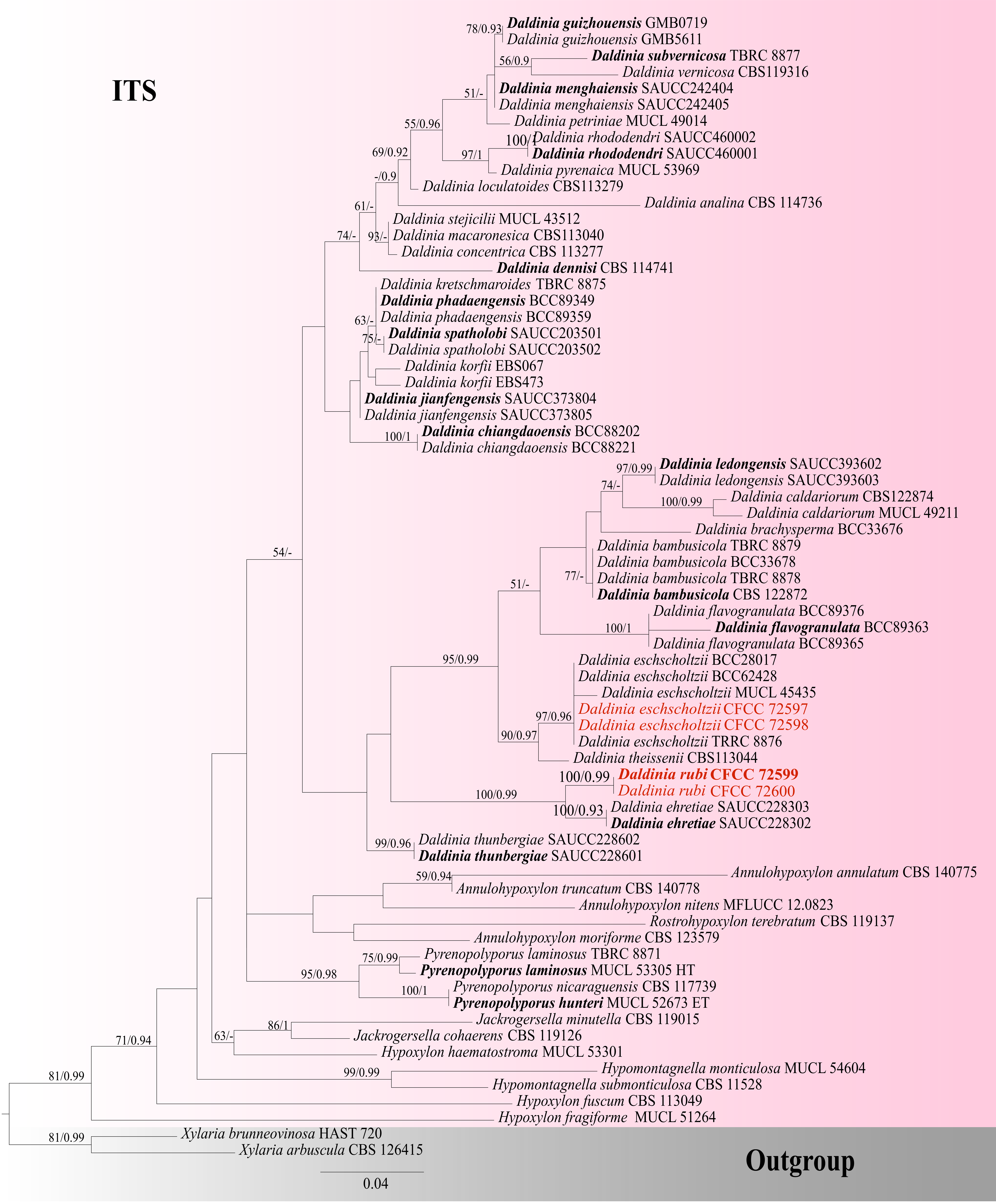

Figure S1-1. Phylogram generated from RAxML analysis based on ITS sequence data of *Daldinia* isolates. The ML (≥ 50%) and BI (≥ 0.9) bootstrap supports are given near the nodes, respectively.

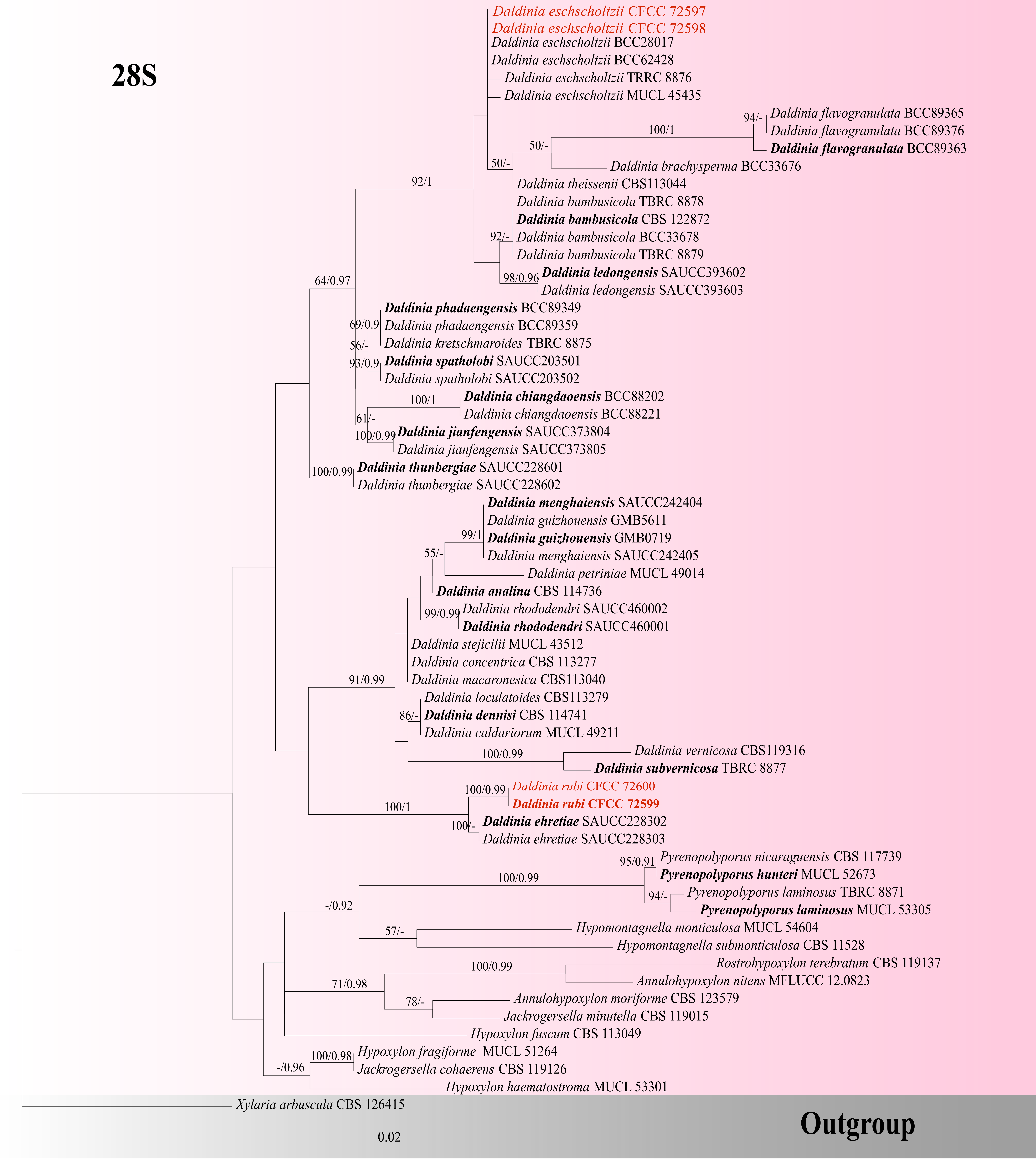

Figure S1-2. Phylogram generated from RAxML analysis based on 28S sequence data of *Daldinia* isolates. The ML (≥ 50%) and BI (≥ 0.9) bootstrap supports are given near the nodes, respectively.

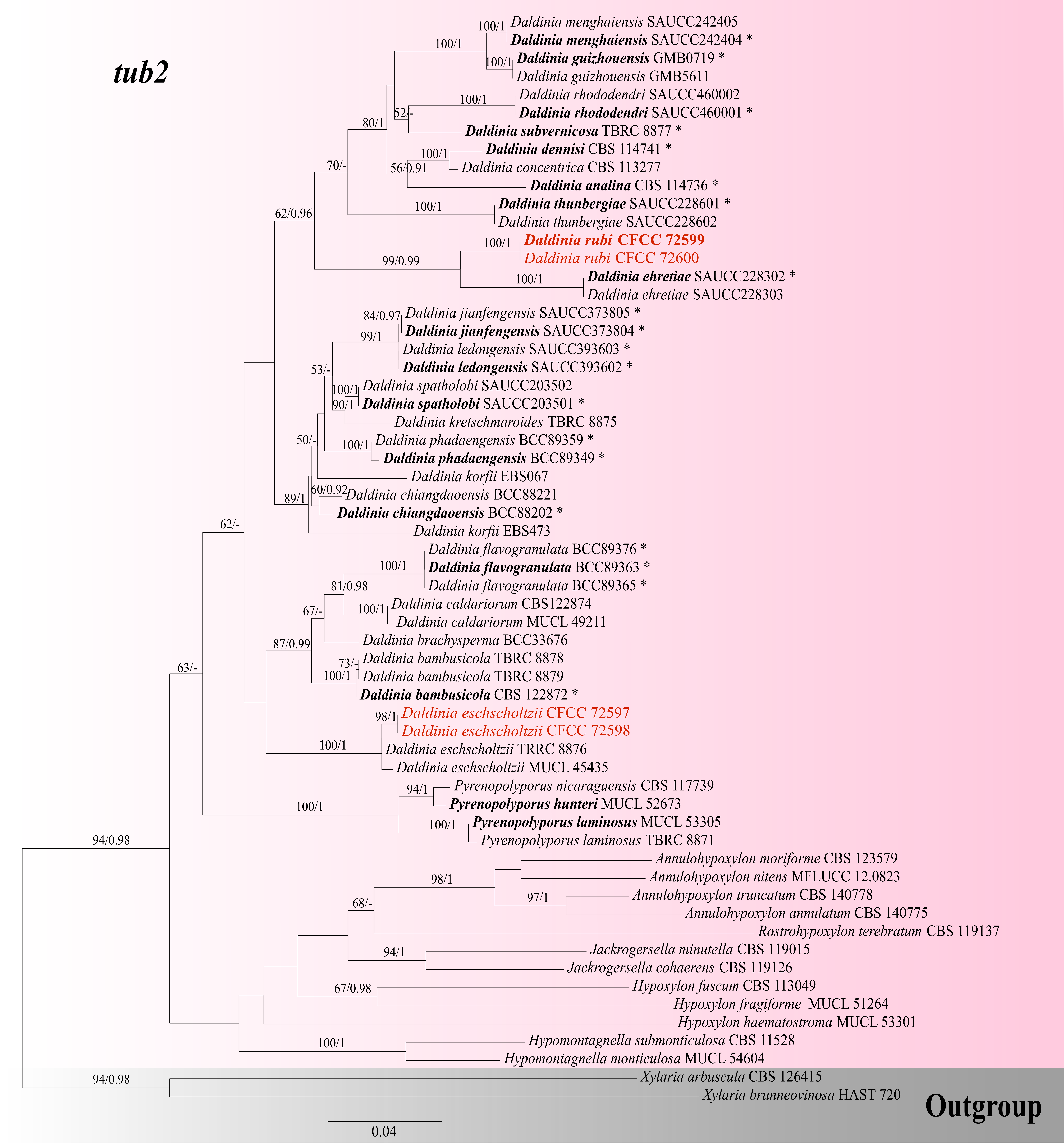

Figure S1-3. Phylogram generated from RAxML analysis based on tub2 sequence data of *Daldinia* isolates. The ML (≥ 50%) and BI (≥ 0.9) bootstrap supports are given near the nodes, respectively.
