# Supplementary material for: ﻿Morphological and molecular identification of new species and records of Daldinia (Hypoxylaceae, Xylariales) from Guizhou Province, China
Source: MycoKeys. 2025 Oct 16;123:253–69. doi: 10.3897/mycokeys.123.160960 (PMC12550505; doi:10.3897/mycokeys.123.160960)
Supplement: Supplementary material 2 — Supplementary table [file mycokeys-123-253-s002.docx]

**Supplementary Table 1.** Host range of *Daldinia* *eschscholtzii*.

| **Species** | **Host Family** | **Host Species** | **Country** |
| --- | --- | --- | --- |
| *Daldinia eschscholtzii* | Acanthaceae | *Graptophyllum pictum* | China |
|  | Altingiaceae | Liquidambar sp. | Mexico |
|  |  | *Liquidambar formosana* | China |
|  | Anacardiaceae | *Mangifera indica* | Cook Islands; China; Seychelles |
|  |  | *Rhus chinensis* | China |
|  | Annonaceae | *Annona muricata* | Sierra Leone |
|  | Apiaceae | *Heteromorpha trifoliata* | Unknown |
|  | Apocynaceae | *Tabernaemontana divaricata* | China |
|  | Arecaceae | *Roystonea regia* | Cuba |
|  | Asparagaceae | *Cordyline fruticosa* | China |
|  | Asphodelaceae | *Aloe vera* | Bangladesh |
|  | Asteraceae | *Ageratina adenophora* | China |
|  |  | *Tithonia diversifolia* | China |
|  | Betulaceae | *Betula utilis* | China |
|  | Boraginaceae | Cordia sp. | Ethiopia |
|  | Burseraceae | *Bursera simaruba* | Unknown |
|  | Cannabaceae | *Celtis kraussiana* | Unknown |
|  | Casuarinaceae | Casuarina sp. | Malaysia |
|  | Combretaceae | *Bucida palustris* | Cuba |
|  | Crassulaceae | *Bryophyllum pinnatum* | India |
|  | Dipterocarpaceae | *Parashorea chinensis* | China |
|  | Dryopteridaceae | *Dryopteris podophylla* | China |
|  | Elaeocarpaceae | *Elaeocarpus decipiens* | China |
|  | Ericaceae | *Rhododendron latoucheae* | China |
|  | Euphorbiaceae | *Croton* sp. | Unknown |
|  |  | Hevea sp. | Indonesia |
|  |  | *Hevea brasilensis* | Philippines; Singapore |
|  |  | *Hevea brasiliensis* | Ghana |
|  |  | *Hippomane manchinella* | Brazil |
|  |  | *Triadica cochinchinensis* | China |
|  | Fabaceae | *Albizia zygia* | Ghana; Sierra Leone |
|  |  | Albizzia sp. | Bahamas; Western Samoa |
|  |  | *Calliandra haematocephala* | China |
|  |  | *Cassia floribunda* | Kenya |
|  |  | *Piscidia piscipula* | Mexico |
|  |  | *Tamarindus indica* | Philippines; Zambia |
|  | Fagaceae | *Castanopsis calathiformis* | China |
|  |  | *Lithocarpus henryi* | China |
|  |  | Quercus sp. | India; United States |
|  |  | *Quercus acutissima* | Indonesia; Japan |
|  | Hamamelidaceae | *Exbucklandia populnea* | China |
|  | Iridaceae | *Iris tectorum* | China |
|  | Juglandaceae | *Engelhardiaspicata* | China |
|  | Lamiaceae | *Pogostemon cablin* | China |
|  | Lauraceae | *Cinnamomum porphyrium* | Argentina; Bolivia |
|  |  | *Lindera aggregata* | China |
|  |  | *Lindera nacusua* | China |
|  |  | *Litsea cubeba* | China |
|  |  | *Machilus nanmu* | China |
|  |  | *Phoebe zhennan* | China |
|  | Malvaceae | *Tilia cordata* | China |
|  | Meliaceae | *Entandrophragma cylindrum* | Democratic Republic of the Congo |
|  |  | *Trichilia minutiflora* | Mexico |
|  | Moraceae | *Artocarpus altilis* | Ghana; Nigeria |
|  |  | *Artocarpus heterophyllus* | China |
|  |  | *Artocarpus hypargyreus* | China |
|  |  | *Artocarpus integrifolia* | Bangladesh |
|  |  | *Brosimum alicastrum* | Mexico |
|  |  | Ficus sp. | Ethiopia; Thailand;Guadeloupe |
|  |  | *Ficus subulata* | China |
|  |  | *Ficus tinctoria* | China |
|  |  | *Morus alba* | Pakistan |
|  | Musaceae | *Musa paradisiaca* | Unknown |
|  |  | *Musa* sp. | Thailand. |
|  | Myrtaceae | *Jambosa vulgaris* | Cuba |
|  |  | *Psidium cattleyanum* | China |
|  |  | *Psidium guajava* | India |
|  |  | *Sizygium jambos* | Cuba |
|  |  | *Syzygium levinei* | China |
|  |  | *Syzygium polyanthum* | Indonesia |
|  | Nothofagaceae | *Nothofagus* sp. | New Zealand |
|  | Oleaceae | *Chengiodendron matsumuranum* | China |
|  | Orchidaceae | *Dendrobium chrysotoxum* | Unknown |
|  | Phyllanthaceae | *Bischofia javanica* | China |
|  |  | *Bridelia balansae* | China |
|  | Pinaceae | Pinus sp. | Cuba |
|  | Piperaceae | *Piper nigrum* | China |
|  | Platanaceae | *Platanus wrightii* | United States |
|  | Poaceae | *Dendrocalamus latiflorus* | China |
|  |  | *Fargesia spathacea* | China |
|  |  | *Microstegium vimineum* | China |
|  |  | *Pseudosasa japonica* | China |
|  | Primulaceae | *Lysimachia clethroides* | China |
|  |  | *Maesa japonica* | China |
|  | Ranunculaceae | *Clematis uncinata* | China |
|  | Rosaceae | *Rhaphiolepis indica* | China |
|  | Rubiaceae | *Diplospora dubia* | China |
|  | Rutaceae | Citrus sp. | United States; Federated States of Micronesia |
|  |  | *Citrus × aurantium* | Cuba |
|  |  | *Citrus aurantifolia* | Ghana; Western Samoa |
|  |  | *Citrus aurantium* | India; Western Samoa |
|  |  | *Citrus limon* | India |
|  |  | *Citrus nobilis* | Brunei Darussalam |
|  |  | Citrus nobilisvar. tangerina | Brazil |
|  |  | Citrus stump | Mexico; Puerto Rico |
|  |  | *Murraya exotica* | China |
|  | Sabiaceae | *Meliosma rigida* | China |
|  | Sapindaceae | *Gonocaryum lobbianum* | China |
|  | Schisandraceae | *Kadsura longipedunculata* | China |
|  | Solanaceae | *Lycianthes biflora* | China |
|  | Theaceae | *Camellia japonica* | South Korea |
|  |  | *Camellia oleifera* | China |
|  |  | *Eurya groffii* | China |
|  |  | *Eurya nitida* | China |
|  |  | *Polyspora chrysandra* | China |
|  |  | *Schima superba* | China |
|  | Thelypteridaceae | *Pronephrium* sp. | China |
|  |  | *Pronephrium gymnopteridifrons* | China |
|  | Zingiberaceae | *Zingiber zerumbet* | China |
